# Motor Training Integrating Cognitive–Behavioral Strategies and Narrative Grammar in Children with Autism Using the Global Integration Method (MIG): A Randomized Clinical Trial

**DOI:** 10.3390/healthcare14142203

**Published:** 2026-07-21

**Authors:** Thalita Karla Flores Cruz, Reinaldo da Costa Paulino Netto, Elisa Braz Cota Fernandes, Amanda Aparecida Alves Cunha Nascimento, Simone Rosa Barreto, Ana Clara de Carvalho Silva, Iolanda Costa Rodrigues, Ana Clara Schaper Fernandes, Gabriela Corrêa Rocha, Deisiane Oliveira Souto

**Affiliations:** 1 Institute of Neurodevelopment, Cognition and Inclusive Education, Belo Horizonte 33900-720, MG, Brazil; thalitacruz@incei.com.br (T.K.F.C.); reinaldopaulino@incei.com.br (R.d.C.P.N.); simonenunes@incei.com.br (S.R.B.); anaclarasilva@incei.com.br (A.C.d.C.S.); iolandarodrigues@incei.com.br (I.C.R.); anaclarafernandes@incei.com.br (A.C.S.F.); gabrielarocha@incei.com.br (G.C.R.); 2 Postgraduate Program in Neuroscience, Federal University of Minas Gerais, Belo Horizonte 31270-901, MG, Brazil; 3 Postgraduate Program in Psychology, Cognition and Behavior, Federal University of Minas Gerais, Belo Horizonte 31270-901, MG, Brazil; elisafernandes@incei.com.br; 4 Graduate Program in Speech-Language Sciences, Federal University of Minas Gerais, Belo Horizonte 31270-901, MG, Brazil; 5 Postgraduate Program in Occupational Sciences, Federal University of Minas Gerais, Belo Horizonte 31270-901, MG, Brazil

**Keywords:** autism, child, motor skills

## Abstract

**Introduction:** A high prevalence of motor deficits has been documented in individuals with Autism Spectrum Disorder (ASD); these deficits negatively impact participation and functional performance. Conventional interventions, which are often fragmented, may limit skill acquisition and generalization. The Global Integration Method (MIG) proposes an integrative approach that combines motor training, proprioceptive stimulation, and cognitive–behavioral strategies mediated through narrative. This study evaluated its effectiveness compared with conventional treatments. **Methods:** This study was designed as a three-arm randomized controlled clinical trial with assessor blinding, including 66 children with ASD aged 6–12 years. Participants were allocated to MIG, conventional physiotherapy, or conventional psychology groups. Interventions were delivered over five weeks. Assessments were performed at baseline, post-intervention, and at a three-month follow-up. Primary outcomes included motor skills (TGMD-2) and functional performance (COPM). Secondary outcomes included balance (PBS) and socio-communicative skills (PROC, ABFW). Analyses followed the intention-to-treat principle using repeated-measures ANOVA. **Results:** The MIG group showed significant improvement compared with control groups in the primary outcomes, with a significant group × time interaction for fundamental motor skills and functional performance (*p* < 0.05), maintained at follow-up. Improvements were also observed in balance and socio-communicative skills (*p* < 0.05). No significant differences were found for adaptive behavior or self-reported motor coordination. Adherence was high, and adverse events were mild. **Conclusions:** MIG was associated with greater improvements in motor and functional outcomes compared with conventional interventions. However, these findings should be interpreted as preliminary evidence of an intensive, multicomponent intervention package, in which treatment dose and combined therapeutic components may jointly contribute to the observed effects.

## 1. Introduction

Autism Spectrum Disorder (ASD) is a neurodevelopmental condition characterized by ongoing difficulties in social communication accompanied by restricted and repetitive behaviors [1]. Although established diagnostic criteria have historically centered on sociocommunicative domains, contemporary evidence indicates that motor skill deficits are highly prevalent in ASD, affecting between 80% and 90% of individuals on the spectrum [2]. Motor difficulties, which range from impairments in postural control to limitations in object coordination, are not isolated comorbidities but are directly related to the individual’s ability to explore the environment and engage in social participation. Consequently, these limitations directly influence the development of more complex cognitive and functional skills [3,4].

Despite the clinical relevance of the motor component, the conventional care model for ASD remains strongly centered on psychobehavioral interventions, such as Applied Behavior Analysis (ABA) and classical neurofunctional physiotherapy [5]. Although effective within their respective domains, these approaches often operate in isolation, which may limit the generalization of learned skills to daily life contexts. Furthermore, conventional motor therapies frequently lack a narrative or play-based structure that promotes engagement and emotional regulation, elements that are crucial for treatment adherence in children and adolescents with ASD.

Given the limitations observed in traditional approaches, there is a growing need for interventions that systematically integrate motor training with proprioceptive stimuli and cognitive–behavioral strategies. In this context, the Global Integration Method (MIG) proposes an interdisciplinary program that combines the use of compressive therapeutic garments (MIG Flex) with motor activities organized through a narrative-based framework, aiming to optimize postural support, motor organization, and child engagement [6,7,8,9]. This proposal is based on the hypothesis that proprioceptive stimulation associated with contextualized tasks may promote not only gains in motor capacity, understood as the potential for performance in a structured environment, but also improvements in functional performance in daily life, facilitating the transfer and generalization of skills, in addition to possible indirect effects on balance and social communication [7].

This approach is grounded in contemporary theoretical models, such as predictive coding theory, which postulates that the brain operates through the continuous generation and updating of predictions based on sensory information [10,11]. In addition, it is supported by the perspective of embodied cognition, which understands cognitive and sociocommunicative processes as intrinsically dependent on bodily and motor experiences [12]. In ASD, evidence indicates impairments in sensory integration and in the construction of stable predictive models, compromising behavioral organization and adaptation to the environment. Consequently, greater variability in motor responses and difficulties in action anticipation are frequently observed. Therefore, interventions that promote consistency of bodily signals, postural stability, and predictability of actions may contribute to reducing prediction errors and improving neurofunctional organization [6]. In this sense, the use of resources such as the MIG Flex garment, by promoting body alignment and continuous proprioceptive feedback, may act as a facilitator of these processes [5].

From an operational perspective, MIG differs by structuring the intervention within highly organized environments, such as the “City of Tomorrow,” which simulate real-life daily contexts and promote situated learning as well as the immediate generalization of skills [6]. In addition, it systematically incorporates cognitive–behavioral strategies and elements of narrative grammar, providing predictability, coherence, and support for information processing, aspects that are particularly relevant for children with ASD [12]. The importance of narrative grammar for the organization of thought, action, and learning is highlighted in the MIG protocol study [9]. Furthermore, family-centered therapeutic planning guided by individualized functional goals reinforces the clinical applicability of the method, aligning therapeutic objectives with the real-life demands of participation and performance in everyday activities [13].

Although MIG presents a consistent theoretical foundation and preliminary evidence of functional, communicative, and motor improvement in children and adolescents with ASD [5,7,8], particularly in outcomes sensitive to change and in parental perceptions, these findings are still incipient. Important gaps remain in the literature regarding the existence of randomized clinical trials that rigorously evaluate its effectiveness in comparison with conventional standard interventions, such as motor physiotherapy and cognitive–behavioral psychological approaches. In this context, the production of robust evidence is essential to support clinical decision-making, optimize the allocation of therapeutic time, expand the achievement of functional goals, and strengthen family-centered practices. Therefore, conducting experimental studies investigating the effects of MIG in direct comparison with conventional treatments is justified. Because treatment intensity may influence rehabilitation outcomes independently of intervention content, the present study evaluates MIG as an integrated intervention package rather than attempting to isolate the effects of its individual components.

This randomized clinical trial aims to examine whether the MIG program contributes to improvements in fundamental motor skills (capacity) and facilitates the accomplishment of functional goals in children and adolescents with ASD, compared with conventional interventions, including motor physiotherapy and psychological approaches developed according to cognitive–behavioral approaches and Applied Behavior Analysis principles. Secondary analyses will explore the effects of MIG on balance, sociocommunicative functioning, and motor performance.

## 2. Methodology

### 2.1. Study Design and Ethics

This was a three-arm randomized controlled clinical trial with assessor blinding whose protocol had been previously published, including a detailed description of the methodological procedures [9]. The study was conducted in outpatient rehabilitation services in the municipality of Ribeirão das Neves. Participants were randomly allocated into three groups, with appropriate allocation concealment, and outcome assessors remained blinded to group assignment. Assessments were performed at three time points: baseline, immediately following the intervention, and three months after the end of the intervention period. The trial was reported in accordance with the Consolidated Standards of Reporting Trials (CONSORT) guidelines. Ethical approval was granted by the Research Ethics Committee of Faculdade de Ciências Médicas de Minas Gerais (approval No. 7.456.658; 28 July 2025). The study was prospectively registered in the Brazilian Registry of Clinical Trials (ReBEC; RBR-7r6n8zd) and in the World Health Organization Universal Trial Number system (UTN: U1111-1326-2272), with registration published on 9 December 2025. Participant recruitment and data collection started only after completion of the registration process. Written informed consent was provided by the participants’ legal guardians, and assent was obtained from the children.

### 2.2. Participants

A convenience sample was recruited from public institutions, philanthropic organizations, and private physiotherapy clinics according to recruitment feasibility and access to the target population. Children aged 6 to 12 years with a previous clinical diagnosis of Autism Spectrum Disorder (ASD) and classified as support levels I or II were considered eligible. ASD diagnoses had been established by qualified physicians according to the Diagnostic and Statistical Manual of Mental Disorders, Fifth Edition, Text Revision (DSM-5-TR), and medical reports confirming the diagnosis were reviewed during participant screening before enrollment. Participants were permitted to continue any usual educational or healthcare services throughout the study, and no restrictions were imposed regarding concurrent therapies. However, at the time of enrollment, none was receiving regular rehabilitation therapies because they were awaiting access to treatment, primarily through public healthcare waiting lists. Consequently, no concurrent rehabilitation interventions were provided during the study period. Children presenting cognitive, behavioral, or clinical comorbidities that could compromise task comprehension or the safe performance of the interventions were excluded.

### 2.3. Sample Size

The required sample size was estimated at 66 participants, with 22 allocated to each group, based on data from a previous study evaluating the effects of the MIG program in children with ASD [5]. The estimation assumed an effect size of 0.87 obtained from the Canadian Occupational Performance Measure (COPM), statistical power of 80%, a significance threshold of 5%, and an anticipated dropout rate of 20%. Sample size estimation was performed using the G*Power software version 3.1 (Heinrich Heine University Düsseldorf, Düsseldorf, Germany). The COPM was considered the primary outcome for sample size calculation.

### 2.4. Blinding and Randomization

**Figure 1:**
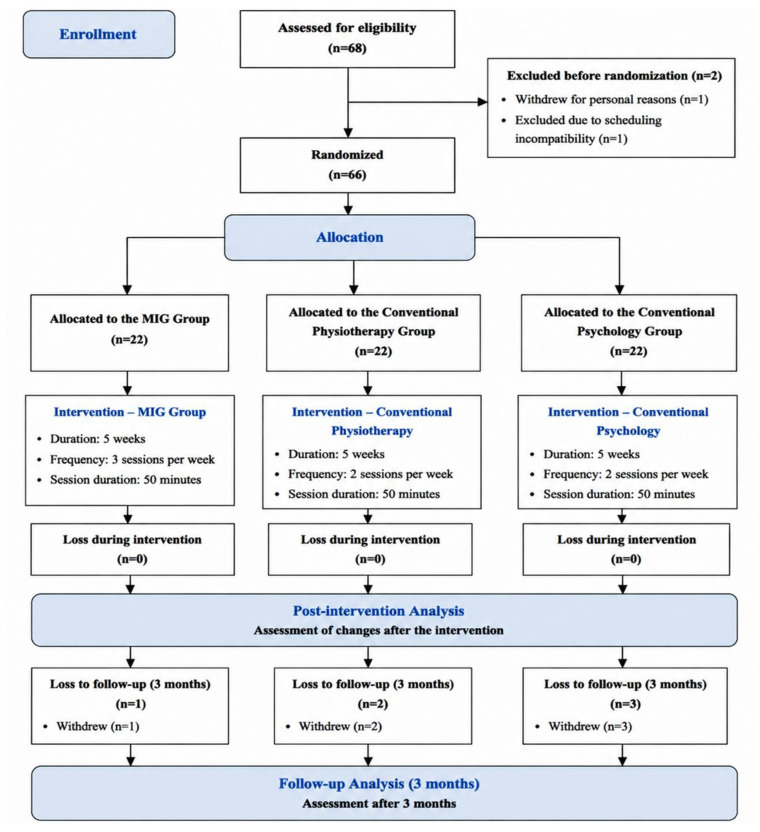
Study Flowchart.

Randomization was performed individually using a computer-generated sequence prepared by an independent researcher who was not involved in the assessments or interventions. Allocation concealment was ensured through opaque, sealed, and sequentially numbered envelopes, which were opened only immediately before the first session. All baseline assessments were conducted prior to randomization. Outcome assessors remained blinded to participant allocation throughout the study. Blinding of participants and intervention providers was not feasible owing to the nature of the intervention. Outcome assessors remained blinded throughout all assessment time points. Procedures were adopted to minimize disclosure of treatment allocation during evaluations. Figure 1 presents the flow of participants throughout the study.

### 2.5. Interventions

The comparison groups were selected to represent conventional rehabilitation models routinely available in clinical practice. Accordingly, each intervention reflected its usual discipline-specific approach. In contrast, MIG was evaluated as an integrated interdisciplinary rehabilitation program. Therefore, the objective of this trial was to compare different rehabilitation models as implemented in routine practice rather than to isolate the contribution of individual therapeutic components.

#### 2.5.1. MIG Program

Participants in the MIG group underwent a protocol directed toward the motor component of the method, delivered by trained physiotherapists with support from an interdisciplinary team to ensure standardization. The intervention lasted five weeks, with three weekly sessions of 50 min each. The MIG intervention was delivered three times per week according to the original program structure and was intended to represent an intensive rehabilitation model. Accordingly, treatment frequency was considered part of the intervention package rather than an isolated procedural feature. However, because intervention intensity is recognized as an important determinant of rehabilitation outcomes, the observed effects should be interpreted as reflecting the combined influence of treatment dose and the multiple therapeutic components of MIG (including motor training, proprioceptive stimulation, narrative structure, and cognitive–behavioral strategies). Details of MIG program and conventional interventions can be found in the published protocol of the study [9].

During the sessions, the MIG Flex therapeutic garment was used to provide postural support and proprioceptive stimulation. Activities were organized into four stages (movement preparation, core strengthening, motor skills circuit, and contextualized activity). Motor activities were structured narratively, using a story in each session that not only taught but also guided the progression of tasks and movements. The incorporation of narratives enabled the integration of motor activities with psychoeducational aspects, particularly regarding emotions.

The protocol addressed multiple physical capacities, with a gradual increase in complexity, accompanied by positive feedback and strategies such as modeling, prompting, task adaptation, and visual supports. To promote consolidation and generalization of the acquired skills, home-based activities were suggested. The intervention combined cognitive and behavioral strategies focused on motor organization and the functional transfer of learning.

#### 2.5.2. Conventional Physiotherapy

Participants allocated to this group underwent conventional neurofunctional physiotherapy, corresponding to the usual motor rehabilitation approach offered in clinical practice settings. The intervention was administered over a five-week period, comprising two sessions per week lasting 50 min each. A semi-structured session format was adopted, including a warm-up phase (5 min), motor training activities (20 min), balance exercises (10 min), ball-related activities (10 min), and a closing phase (5 min), while allowing therapists to adapt procedures according to individual participant requirements.

This intervention did not involve therapeutic garments, narrative-based elements, cognitive approaches, or intentionally integrated interdisciplinary strategies. All sessions took place in standard therapy rooms at Clínica Reabilitar.

#### 2.5.3. Conventional Psychology

Participants in this group received an intervention delivered by psychology professionals using cognitive and behavioral approaches and principles of Applied Behavior Analysis (ABA), corresponding to standard psychological care. The program was implemented over a five-week period, consisting of two 50 min sessions per week, for a total of 10 sessions.

The protocol emphasized the development of emotional regulation abilities, including recognition of basic emotions (happiness, sadness, fear, disgust, anger, and anxiety) and their associated expressions, identification of emotions in oneself and in others, and the use of strategies to support appropriate emotional expression and regulation.

Sessions were conducted according to a structured therapeutic framework while allowing clinical adaptation to participants’ individual needs. The intervention did not include direct motor training or explicit goals related to motor function. Narrative elements were not adopted as a central therapeutic resource. All sessions took place in standard treatment rooms at Clínica Reabilitar.

### 2.6. Outcomes

#### 2.6.1. Primary Outcomes

The Test of Gross Motor Development—Second Edition (TGMD-2) [14] was employed to evaluate fundamental motor skills. This standardized assessment provides both quantitative and qualitative measures across 12 motor tasks, categorized into locomotor and object control domains [15]. Performance scores are calculated by converting raw scores into motor quotients, enabling normative interpretation of results. Evidence of validity and reliability for the Brazilian typically developing population was reported by Valentini et al. [16], and the instrument has subsequently been applied in research involving children with ASD [5].

The Canadian Occupational Performance Measure (COPM) [17] was applied to evaluate achievement of functional goals. This semi-structured interview enables parents to identify, describe, and prioritize therapeutic goals related to the child’s occupational performance. Each selected goal is scored on a 10-point scale according to perceived importance, performance, and satisfaction. The COPM has demonstrated adequate validity, reliability, and responsiveness to clinical change [17,18].

#### 2.6.2. Secondary Outcomes

Functional balance was assessed using the Pediatric Balance Scale (PBS). The PBS includes 14 items designed to evaluate tasks of increasing complexity that simulate essential activities of daily living and involve both static and dynamic balance demands [19]. Items are scored on a scale from 0 to 4, generating a total score ranging from 0 to 56, with higher values reflecting superior functional balance performance. The instrument has demonstrated adequate psychometric properties for the Brazilian population [19].

Functional communicative abilities were evaluated using the ABFW Pragmatics Test—Child Language Test. Based on systematic observation of spontaneous interactions, this assessment enables both quantitative and qualitative examination of communicative acts, communicative modalities, and communicative functions and has shown sensitivity for assessing pragmatic language performance [20,21].

Communicative and cognitive abilities were assessed using the Behavioral Observation Protocol (PROC) through a structured interaction based on play activities. This instrument allows characterization of language development and symbolic play and has proven particularly useful for identifying patterns of cognitive–communicative functioning in children with developmental conditions [22,23].

Gross and fine motor abilities were evaluated using the motor domain of the Vineland Adaptive Behavior Scales (VABS), administered through parent report [24]. The VABS-II version demonstrates satisfactory psychometric performance, including concurrent validity of 0.77 in comparison with the Peabody Developmental Motor Scales [25].

The Developmental Coordination Disorder Questionnaire (DCDQ) was employed as a parent-reported screening measure to identify motor coordination difficulties in everyday activities. The questionnaire comprises 15 items rated on a 5-point Likert scale and provides age-specific cut-off values to identify children at risk for developmental coordination disorder [26]. Evidence supports its validity and reliability, and the instrument has been culturally adapted and validated for use in Brazil.

### 2.7. Procedures

Outcome assessments were performed by four independent evaluators who remained blinded to group allocation (a physiotherapist, a psychologist, a speech–language pathologist, and an occupational therapist), according to the professional expertise required for each assessment instrument. The physiotherapist administered the PBS and TGMD-2; the occupational therapist conducted the COPM and DCDQ through parent interviews; the psychologist administered the VABS, also based on parent interviews; and the speech–language pathologist conducted the PROC and ABFW assessments. Evaluations were scheduled at three predefined time points: baseline, immediately following the intervention period, and three months after intervention completion. Baseline measurements were obtained before random allocation.

### 2.8. Data Analysis

Statistical analyses were performed using IBM SPSS Statistics Version 26 (IBM SPSS Statistics, NY, USA). Initially, descriptive analyses were conducted to characterize the sample in terms of demographic and clinical variables. Data normality was assessed using the Shapiro–Wilk test. Baseline group comparisons were performed using one-way analysis of variance (ANOVA) or the Kruskal–Wallis test, depending on data distribution.

To evaluate intervention effects over time, repeated-measures analysis of variance (ANOVA) was performed considering group (MIG, conventional physiotherapy, and conventional psychology) as the between-subject factor and time (baseline, post-intervention, and follow-up) as the within-subject factor. Interaction effects between group and time were examined to identify differential changes across interventions.

When significant main effects or interactions were identified, post hoc pairwise comparisons with Bonferroni correction were performed. Effect sizes were expressed as partial eta squared (η^2^p). For relevant between-group comparisons, mean differences and corresponding 95% confidence intervals (95% CI) were reported to improve interpretation of treatment effects.

Sphericity assumptions were assessed using Mauchly’s test, and Greenhouse–Geisser correction was applied when assumptions were violated.

Missing follow-up data were handled according to the intention-to-treat principle using the Last Observation Carried Forward (LOCF) method, whereby the last available observation was carried forward to subsequent missing time points. The effectiveness of assessor blinding was evaluated descriptively at study completion. Statistical significance was set at *p* < 0.05 (two-tailed).

### 2.9. Ethical Aspects

Ethical approval was granted by the Research Ethics Committee of the Faculdade de Ciências Médicas de Minas Gerais (approval No. 7.456.658). The study was prospectively registered in the Brazilian Clinical Trials Registry (RBR-7r6n8zd; identifier U1111-1326-2272).

Written informed consent was provided by parents or legal guardians, and assent was obtained from participants whenever appropriate according to their developmental stage before initiation of any study-related procedures. All study procedures complied with national and international ethical guidelines governing research involving human participants. Adverse events were systematically monitored and documented throughout the intervention period, and participants received appropriate clinical referral whenever indicated.

## 3. Results

A total of 68 participants were initially recruited for the study. Of these, there was a pre-intervention attrition: two participants were excluded at the request of their guardians (one due to personal reasons and the other due to scheduling incompatibility), resulting in a final sample of 66 children who completed the experimental protocol. After randomization, no further losses were recorded during the intervention period in any group. However, at follow-up, six participants (9.06%) were lost to follow-up: one from the MIG group, two from the conventional physiotherapy group, and three from the conventional psychology group. Missing data were handled using the LOCF method, ensuring inclusion of all randomized participants in the analyses according to the intention-to-treat principle.

The groups were homogeneous regarding age (MIG: 8.05 ± 1.79; conventional physiotherapy: 7.86 ± 1.36; conventional psychology: 8.64 ± 2.46; ANOVA: F = 0.972; *p* = 0.384). Sex distribution was similar across groups (male: MIG 18, physiotherapy 17, psychology 17), with no significant association between sex and treatment group (χ^2^ = 0.181; *p* = 0.913). The groups were also homogeneous regarding level of support (Level 1: MIG 11, physiotherapy 13, psychology 9; Level 2: MIG 9, physiotherapy 7, psychology 13), with no significant association between support level and treatment group (χ^2^ = 2.481; *p* = 0.289). Baseline demographic and clinical characteristics demonstrated satisfactory comparability across the three intervention groups. All participants had a previous medical diagnosis of Autism Spectrum Disorder established according to the DSM-5-TR diagnostic criteria. Baseline cognitive functioning, language performance, motor performance, balance, adaptive behavior, communication, and occupational performance were assessed using the standardized outcome measures employed in this study. No statistically significant between-group differences were observed for any baseline outcome measure, indicating that randomization successfully produced comparable groups prior to the intervention. Medication use was also similarly distributed across the groups (MIG: 86.4%, Motor Physiotherapy: 86.4%, Psychology: 63.6%), with no statistically significant between-group differences (Pearson’s χ^2^ = 4.533, *p* = 0.104; Fisher–Freeman–Halton exact test, *p* = 0.135), further supporting the baseline homogeneity of the sample.

Participants in the MIG group demonstrated high adherence, with 86.67% attendance across the 15 planned sessions. Three participants reported mild adverse events, characterized by transient discomfort associated with initial use of the MIG Flex garment. This discomfort decreased progressively during the first session and did not reoccur in subsequent sessions.

In the conventional physiotherapy group, attendance was 86.67%, while in the conventional psychology group it was 93.33%. Throughout the intervention period, no adverse events were documented in these groups.

### 3.1. Primary Outcomes

Table 1 presents the comparison of outcomes across groups, explicitly distinguishing main effects, interaction effects, and within-group post hoc changes over time. For the TGMD-2 Total score, a significant main effect of time was observed (F(2, 108) = 12.774, *p* < 0.001; η^2^p = 0.191), together with a significant group × time interaction (F(4, 108) = 3.472, *p* = 0.012; η^2^p = 0.114), indicating that the magnitude of change over time differed between groups. Post hoc analyses of within-group trajectories showed significant improvements in the MIG group from T0 to T1 and T2 (T1–T0 = 10.53; T2–T0 = 10.11), whereas no significant change was detected in the Psychology group across time points. In contrast, the Physiotherapy group also showed a significant within-group improvement at T2 compared with baseline (T2–T0 = 6.79), indicating that improvements were not exclusive to MIG, although they were larger and more consistent in this group.

For the TGMD-2 Object Control subscale, significant main effects of time (F(2, 108) = 16.185, *p* < 0.001; η^2^p = 0.231) and a group × time interaction (F(4, 108) = 3.043, *p* = 0.024; η^2^p = 0.101) were identified. Within-group post hoc comparisons indicated significant gains in all groups, although with different patterns over time: the MIG group showed improvements both at T1 and T2 relative to baseline, while the Physiotherapy group demonstrated significant improvements both from baseline to follow-up and between intermediate time points, and the Psychology group showed improvements at later time points compared with baseline. These findings indicate that all groups improved over time, but the trajectories and magnitude of change differed, as reflected by the significant interaction effect. For the TGMD-2 Locomotion subscale, a significant main effect of time (F(2, 108) = 3.457, *p* = 0.039; η^2^p = 0.060) and a significant group × time interaction (F(4, 108) = 3.335, *p* = 0.016; η^2^p = 0.110) were observed. Post hoc analyses showed that the MIG group improved significantly from T0 to T1 and T2, whereas the Psychology and Physiotherapy groups did not show statistically significant within-group changes across time points. This pattern suggests that locomotor gains over time were primarily driven by the MIG intervention, consistent with the interaction effect.

For COPM outcomes, both Performance and Satisfaction showed significant main effects of time (Performance: F(1.737, 79.906) = 34.30, *p* < 0.001; η^2^p = 0.427; Satisfaction: F(2, 92) = 31.43, *p* < 0.001; η^2^p = 0.406) and significant group × time interactions (Performance: F(3.474, 79.906) = 4.608, *p* = 0.002; η^2^p = 0.167; Satisfaction: F(4, 92) = 3.92, *p* = 0.007; η^2^p = 0.145). Within-group analyses indicated that the MIG group exhibited significant improvements from baseline to all subsequent time points in both domains. The Physiotherapy group also demonstrated significant within-group gains in COPM Performance and Satisfaction at T2 compared with baseline, while the Psychology group showed no statistically significant changes over time. Overall, these results indicate a general improvement over time, with more robust and consistent within-group gains in the MIG group and selective improvements in one control group depending on the outcome.

### 3.2. Secondary Outcomes

The analysis of the Pediatric Balance Scale (PBS) revealed a significant main effect of time (F(1.726, 81.130) = 5.832, *p* = 0.006; η^2^p = 0.110), as well as a significant time × group interaction (F(3.452, 81.130) = 2.531; *p* = 0.045; η^2^p = 0.097). Post hoc analyses indicated that the MIG group showed significant improvements from baseline (T0) to both post-intervention (T1) and follow-up (T2). In contrast, the control groups (Psychology and Physiotherapy) did not exhibit statistically significant changes over time. These findings suggest that, although temporal changes were present across the sample, only the MIG group demonstrated a consistent and significant improvement in balance performance over time (Table 2).

For the Vineland Adaptive Behavior Scales (VABS-II), no significant main effect of time (F(2, 98) = 0.710; *p* = 0.533; η^2^p = 0.007) or time × group interaction (F(4, 98) = 0.149; *p* = 0.963; η^2^p = 0.006) was observed, indicating that adaptive behavior remained stable over time and followed a similar trajectory across all groups, with no evidence of differential intervention effects.

Similarly, for the DCDQ, there was no significant main effect of time (F(2, 114) = 0.632; *p* = 0.533; η^2^p = 0.011) and no significant time × group interaction (F(4, 114) = 1.142; *p* = 0.340; η^2^p = 0.039), indicating that changes in motor coordination-related outcomes were not statistically significant and did not differ between groups over time.

For expressive language (PROC), a significant main effect of time was observed (F(1.346, 67.305) = 29.077; *p* < 0.001; η^2^p = 0.368), along with a significant time × group interaction (F(2.692, 67.305) = 3.214; *p* = 0.033; η^2^p = 0.114), indicating differential changes across groups over time. Post hoc comparisons showed that the MIG group demonstrated significant improvements from T0 to T1, which were maintained at T2. The Physiotherapy group also showed significant improvements over time, with increases from baseline to both post-intervention and follow-up assessments. The Psychology group did not show statistically significant changes over time. These results indicate that improvements in expressive language were not exclusive to the MIG group but varied according to intervention type. For verbal comprehension (PROC), a significant main effect of time was found (F(1.470, 73.510) = 7.184; *p* = 0.004; η^2^p = 0.126), whereas no significant time × group interaction was observed (F(2.940, 73.510) = 1.225; *p* = 0.306; η^2^p = 0.047). This pattern indicates that all groups, on average, improved over time in a similar.

Finally, analysis of total communicative acts (ABFW) showed a significant main effect of time (F(2, 102) = 19.634; *p* = 0.001; η^2^p = 0.278) and a significant time × group interaction (F(4, 102) = 3.757; *p* = 0.007; η^2^p = 0.128). Post hoc analyses indicated that the MIG group exhibited significant increases from T0 to T1, which were maintained at T2. The control groups did not demonstrate statistically significant changes over time. These results suggest that improvements in communicative behavior were more pronounced and consistent in the MIG group compared to the control interventions.

## 4. Discussion

In this randomized clinical trial involving 66 children with ASD, the effectiveness of the MIG program was investigated with respect to improvements in fundamental motor skills and functional goal achievement, compared with conventional motor physiotherapy and psychological interventions grounded in cognitive–behavioral techniques and Applied Behavior Analysis. The study also investigated effects on balance, sociocommunicative skills, and motor performance.

The results demonstrated that the MIG program was associated with greater improvements within the context of the evaluated intervention package in both motor capacity, as measured by the TGMD-2, and functional performance and parental satisfaction, assessed by the COPM, with maintenance of effects over a three-month follow-up period. Additionally, relevant improvements were observed in functional balance and in specific domains of expressive language and communicative acts, which raise the hypothesis that motor organization and postural control may be associated with changes in sociocommunicative engagement. However, these interpretations remain speculative, as no mediation analyses were conducted. In contrast, no significant between-group differences were found for adaptive behavior and parent-reported motor coordination, which may indicate the need for longer intervention periods to modify these constructs. The main findings are discussed below in light of the current literature.

An important consideration when interpreting these findings is that the MIG group received a higher treatment frequency than the comparison groups. Because treatment intensity is an integral component of the original MIG program, this trial was designed to evaluate the effectiveness of the intervention as a multicomponent rehabilitation package rather than the isolated contribution of its individual therapeutic components. In addition to motor training, MIG incorporates proprioceptive stimulation through the MIG Flex garment, cognitive–behavioral strategies, narrative grammar, and an intensive treatment schedule. Consequently, it is not possible to determine the relative contribution of the additional weekly session to the observed outcomes or to disentangle its effects from those of the other intervention components. Therefore, the findings should be interpreted as reflecting the combined effects of treatment intensity and the integrated multicomponent nature of the MIG program, rather than any single therapeutic element. This perspective is consistent with rehabilitation research in which complex interventions are evaluated as integrated therapeutic programs, although dismantling studies are required to determine the contribution of each component.

The findings of this study indicate that the MIG-based intervention promoted significant improvements in global motor performance and its specific domains (object control and locomotion) when compared with control groups. These results are consistent with existing literature on task-oriented and intensive practice approaches [27,28]. The observed improvements may be related to features such as task-oriented practice, variability of motor experiences, and structured functional activities, which enhance active child engagement. These elements are widely recognized in the literature as important for motor learning, as they may support mechanisms such as experience-dependent neuroplasticity and refinement of motor patterns [27,28].

In addition, MIG appears to incorporate principles of intensity and structured repetition, combined with appropriate feedback, which are considered in the literature as important factors associated with the consolidation of new motor skills, in contrast to less structured approaches or those with reduced emphasis on active practice, as possibly observed in the Psychology and Physiotherapy groups. Another relevant aspect involves the integration between sensorimotor systems and cognitive and motivational components, which may support not only execution but also planning and motor adaptation in dynamic tasks. These results are consistent with evidence showing that task-oriented, high-intensity, and ecologically relevant interventions tend to produce greater gains in fundamental motor skills in children [29,30], reinforcing the importance of carefully designing interventions that align components and mechanisms to maximize clinical outcomes.

The improvements observed in COPM Performance and Satisfaction suggest that the benefits of the MIG intervention extended beyond motor skill acquisition to outcomes related to functional goal attainment and caregiver-perceived performance in everyday activities. The higher satisfaction reported by caregivers in the MIG group should be interpreted with caution, as it may reflect not only perceived improvements in the child’s functional abilities but also the greater therapeutic exposure and increased opportunities for therapist–family interaction resulting from the intensive intervention schedule. Nevertheless, caregiver satisfaction represents a clinically meaningful outcome in pediatric rehabilitation, particularly in autism, where family engagement is a central component of intervention and families often face substantial emotional and practical demands. Compared with the conventional interventions evaluated in this study, the MIG program integrates individualized goal setting, meaningful task practice, and active child participation within a structured therapeutic framework that is consistent with the principles of family- and client-centered care. These characteristics may contribute to improvements in occupational performance through factors such as enhanced motivation, engagement, and self-efficacy, which have been associated with positive rehabilitation outcomes in previous studies [31,32]. However, because neither mediation analyses nor dismantling designs were conducted, these explanations should be regarded as plausible hypotheses rather than evidence of the specific mechanisms responsible for the observed effects.

In addition, the use of ecologically valid and contextually meaningful tasks may have contributed to greater transfer of gains into daily life, reflected in both improved performance and perceived satisfaction. The magnitude of the observed effects (high η^2^p values) suggests that the intervention may influence not only the ability to execute tasks but also how these tasks are experienced by the child or adolescent, which is consistent with evidence highlighting that goal-oriented and participation-centered interventions are more effective in promoting clinically meaningful changes in self-reported outcomes [33,34]. Thus, the findings may indicate that the combination of meaning, engagement, and task-oriented practice is relevant to broader dimensions of functionality.

The results of the Pediatric Balance Scale suggest that the gains observed in the MIG group may be partially associated with specific components of the intervention, such as the use of the MIG Flex garment, which may act as a sensory modulator during motor practice. More specifically, this resource may provide increased proprioceptive input and support postural organization, thereby potentially facilitating control of the center of mass during dynamic tasks. This type of stimulus is associated in the literature with improved sensory integration and anticipatory muscle activation, both considered relevant for functional balance.

Evidence indicates that interventions using proprioceptive enrichment strategies and postural challenges promote more efficient adjustments and better stability in pediatric populations with motor impairments [35,36]. Furthermore, studies involving therapeutic garments, such as dynamic orthoses and elastic suits, have demonstrated positive effects on postural alignment and trunk control, which may be related to improvements in balance performance [37,38]. Thus, it is plausible that the combination of functional practice and use of the MIG Flex garment may have contributed to postural control processes, which could be associated with the gains observed exclusively in the MIG group.

The absence of significant differences in the Vineland and DCDQ suggests that the gains observed in more specific outcomes did not translate into detectable changes in adaptive behavior and parent-reported motor coordination in daily life. This finding may be explained by the more distal nature of these outcomes, which depend heavily on the application of skills in real-world contexts and on environmental factors.

In this sense, although MIG included components effective in improving performance in clinical tasks, it is possible that ingredients specifically aimed at generalization were not incorporated with sufficient intensity, such as systematic practice in home and school environments, active caregiver involvement, and adaptation of daily life activities. From a conceptual perspective, transfer of gains to adaptive behavior likely involves not only skill acquisition but also repeated use in ecologically valid and meaningful situations, which may support consolidation and functional use of these competencies.

Evidence indicates that purely clinic-based interventions tend to have limited impact on participation outcomes when not combined with contextual strategies such as parent coaching and environmental modification [34,39]. Thus, these findings highlight the potential importance of integrating real-world environmental actions into therapeutic interventions in order to enhance generalization of gains and promote broader changes in child functioning.

The results related to sociocommunicative skills indicate an interesting pattern of differentiated effects across outcomes. The improvement observed in expressive language in both the MIG group and the physiotherapy group suggests that shared components between interventions, such as structured motor practice and therapeutic interaction in both groups, as well as communicative stimulation through narratives during MIG sessions, may have contributed to verbal production. More specifically, the intervention structure itself, with increased child engagement in guided activities and greater opportunities for communication, may facilitate language development.

On the other hand, the absence of changes in verbal comprehension and cognitive development suggests that these domains, being more complex and less directly trained, may require more specific and prolonged interventions. Language comprehension involves receptive processing, auditory integration, and meaning construction, aspects that were not directly targeted by the interventions. Similarly, cognitive development, as a broader construct, tends to be less sensitive to short-term interventions. For changes in this domain, greater intensity, variability of experiences, and often targeted interdisciplinary approaches are necessary [40].

In contrast, the significant increase in communicative acts observed exclusively in the MIG group suggests a possible association between components of this intervention and nonverbal communicative behaviors, rather than indicating specific underlying mechanisms. The deficit in the ability to plan complex motor sequences (such as those required for speech or symbolic gestures), frequently observed in individuals with autism, is associated with delays in language development and socioemotional reciprocity [41,42]. Since many social and pre-linguistic acts have a motor basis, difficulties in imitation related to dyspraxia may act as a barrier to nonverbal communication [42]. Within this context, a plausible hypothesis is that MIG, by emphasizing motor organization and embodied engagement, may have supported the emergence of nonverbal communicative behaviors, such as gestures, pointing, and intentional bodily expressions.

Another hypothesis is that the use of narratives may have played a crucial role in the increase in communicative acts observed in the MIG group. Narratives offer an organized context that facilitates the understanding and expression of actions and intentions, promoting the structuring of motor and cognitive sequences [43,44]. By engaging participants in stories that involve the representation of actions, emotions, and relationships between characters, MIG may have offered a structured and meaningful context for communicative engagement, potentially favoring communicative initiative, turn-taking, and participation.

The absence of changes in broader outcomes, such as comprehension and cognition, should be interpreted with caution. Preserved oral comprehension was an inclusion criterion for the study, as it was necessary for the motor training, meaning that the children already showed high performance in this domain at baseline. Thus, a ceiling effect may have limited the detection of subtle gains in these measures. Furthermore, although narrative grammar-based interventions may influence receptive skills, the instruments used may not have been sensitive enough to capture small changes in this domain. Overall, these findings suggest that the MIG protocol, primarily focused on motor components, may be more directly related to the emergence of communicative acts and expressive language, whereas broader changes in the sociocommunicative profile may require complementary interventions and more sensitive assessment strategies.

This study presents several important strengths that reinforce the robustness of its findings. First, the experimental design as a three-arm randomized controlled trial with blinded assessors reduces selection and measurement bias, increasing internal validity. Additionally, the prior publication of the study protocol contributes to methodological transparency, reduces reporting bias, and strengthens reproducibility. The use of multiple outcomes encompassing capacity, functional performance, and participation allows for a comprehensive analysis aligned with contemporary models of functioning. Furthermore, the detailed description of MIG components, including motor training, cognitive–behavioral strategies, narrative grammar, and the use of the MIG Flex garment, facilitates replication of the intervention and provides a framework for future studies to investigate the contribution of its individual components and their potential mechanisms of action. The inclusion of follow-up assessment and high participant adherence further reinforce the consistency of the results.

On the other hand, some limitations should be considered. The relatively short intervention period may not have been sufficient to impact more distal outcomes such as adaptive behavior. Additionally, participants allocated to the MIG group received three sessions per week, whereas participants in the conventional physiotherapy and psychology groups received two weekly sessions. This difference reflects the original structure of the MIG program, which was conceived as an intensive intervention model in which treatment frequency constitutes one of its therapeutic components. Consequently, the observed effects should be interpreted as resulting from the intervention package as a whole, including its intensity, rather than being attributed exclusively to specific elements such as narrative grammar, cognitive–behavioral strategies, or the MIG Flex suit. Accordingly, the present study does not allow conclusions regarding the individual contribution of these components or the mechanisms through which they may have influenced the observed outcomes. Future studies comparing interventions with equivalent treatment doses will be important to clarify the relative contribution of treatment intensity and the specific components of the intervention. The lack of blinding of participants and therapists, inherent to the nature of the intervention, may introduce performance bias. In addition, the use of parent-reported measures may limit sensitivity to subtle changes. Finally, although home-based activities were suggested, there was no systematic monitoring of their implementation, which may have limited the generalization of gains to real-life contexts.

The findings of the present study should be interpreted within the context of the current stage of evidence development regarding MIG. Although prior studies have provided preliminary support for the theoretical rationale and potential clinical applicability of this intervention, a considerable proportion of the available literature is still based on early-phase investigations. Accordingly, the existing evidence should be regarded as emerging and requiring confirmation through independent studies conducted in diverse clinical settings and populations.

A relevant limitation of this study concerns the difference in treatment dosage between groups, with participants allocated to the MIG group receiving three sessions per week, whereas the comparison groups received two weekly sessions. This difference reflects the original design of the MIG program, in which treatment intensity constitutes an integral component of a multicomponent rehabilitation package. Consequently, the present study was designed to evaluate the effectiveness of the intervention as implemented in clinical practice rather than to determine the independent contribution of treatment intensity or any other individual therapeutic component. Therefore, the findings should be interpreted as reflecting the combined effects of treatment intensity and the integrated nature of the MIG program, rather than being attributed to specific components in isolation. Future randomized trials employing equivalent treatment frequencies and dismantling or factorial designs will be necessary to distinguish the relative contribution of treatment intensity from that of the individual components of the intervention.

An additional methodological limitation relates to the statistical approach used for handling repeated measurements and missing data. The analysis was performed using repeated-measures ANOVA combined with the Last Observation Carried Forward (LOCF) method. Missing data were limited (9.1%), and follow-up losses were distributed across the three intervention groups, reducing the likelihood that missing data substantially influenced the study conclusions. Although this strategy was pre-specified and is commonly applied in randomized clinical trials with low attrition rates, no additional sensitivity analyses were performed to evaluate the robustness of the findings under alternative missing-data assumptions. Furthermore, repeated-measures ANOVA does not capture individual variability in longitudinal trajectories as flexibly as linear mixed-effects models. Future research should consider the use of mixed-effects modeling together with complementary sensitivity analyses to further strengthen the robustness and precision of longitudinal inferences.

## 5. Conclusions

In summary, this randomized clinical trial demonstrates that the MIG program was associated with greater improvements in fundamental motor skills and functional goal attainment compared with conventional interventions. The findings also point to additional benefits in balance and specific aspects of communication, suggesting that integrative approaches that combine motor, sensory, cognitive, and behavioral components may yield broader and more consistent effects than more fragmented intervention models. Although no significant changes were observed in more distal outcomes, such as adaptive behavior and perceived motor coordination in daily life, this pattern may reflect the complexity and time required for functional generalization beyond the training context, particularly in short-term interventions. Overall, these results support the MIG program as a promising intensive, multicomponent rehabilitation approach, in which the combination of higher treatment dose and integrated therapeutic strategies appears to play a meaningful role in enhancing motor and functional outcomes.

## Figures and Tables

**Table 1:** Comparison between the groups’ primary outcome measures.

Outcome	Group	T0 Mean	T1 Mean	T2 Mean	Effect Time	Group × Time	Pairwise Post Hoc Comparisons over Time *
		(SD)	(SD)	(SD)	*p* (η^2^p)	*p* (η^2^p)	Mean Difference [95% CI]
TGMD-2 Total	MIG	65.68 (13.00)	76.21 (8.37)	75.79 (10.37)	<0.001 (0.191)	0.012 (0.114)	T1–T0: 10.53 (5.23 to 15.82); T2–T0: 10.11 (3.66 to 16.55)
	Psychology	71.89 (11.28)	71.00 (9.55)	75.37 (9.18)			NS
	Physiotherapy	61.53 (17.45)	63.05 (14.96)	68.32 (17.51)			T2–T0: 6.79 (0.35 to 13.23)
TGMD-2 Object Control	MIG	31.68 (7.28)	37.21 (5.75)	37.37 (6.04)	<0.001 (0.231)	0.024 (0.101)	T1–T0: 5.53 (2.13 to 8.92); T2–T0: 5.68 (1.38 to 9.99)
	Psychology	33.84 (6.69)	33.32 (5.74)	39.26 (4.44)			T2–T0: 5.42 (1.12 to 9.72); T2–T1: 5.95 (2.49 to 9.40)
	Physiotherapy	29.58 (9.31)	29.63 (7.36)	33.16 (9.03)			T2–T1: 3.53 (0.07 to 6.98); T1–T0: 4.68 (1.61 to 7.76)
TGMD-2 Locomotion	MIG	34.32 (7.40)	39.00 (3.93)	38.42 (5.31)	0.039 (0.060)	0.016 (0.110)	T2–T0: 4.11 (0.13 to 8.08)
	Psychology	38.05 (6.20)	37.68 (6.06)	36.11 (7.32)			NS
	Physiotherapy	31.95 (9.14)	33.42 (8.36)	35.16 (9.14)			NS
COPM Performance	MIG	3.05 (1.35)	6.49 (1.73)	6.49 (2.29)	<0.001 (0.427)	0.002 (0.167)	T1–T0: 3.43 (2.16 to 4.71); T2–T0: 3.43 (2.11 to 4.75)
	Psychology	3.60 (1.70)	4.87 (1.42)	4.66 (1.51)			NS
	Physiotherapy	3.58 (1.70)	4.79 (1.84)	5.29 (1.85)			T2–T0: 1.71 (0.35 to 3.07)
COPM Satisfaction	MIG	2.91 (1.35)	6.60 (2.18)	6.49 (2.59)	<0.001 (0.406)	0.007(0.145)	T1–T0: 3.69 (2.22 to 5.17); T2–T0: 3.57 (2.13 to 5.02)
	Psychology	3.54 (1.86)	4.91 (1.80)	4.50 (2.06)			NS
	Physiotherapy	3.53 (1.59)	5.10 (2.43)	5.72 (2.29)			T1–T0: 1.57 (0.05 to 3.09); T2–T0: 2.19 (0.70 to 3.68)

* Pairwise comparisons correspond to within-group comparisons across assessment time points and were adjusted using the Bonferroni correction. Legend: TGMD-2: Test of Gross Motor Development—Second Edition; COPM: Canadian Occupational Performance Measure; MIG: Global Integration Method; T0 = baseline; T1 = post-intervention; T2 = 3-month follow-up; NS = non-significant; CI = 95% confidence interval.

**Table 2:** Comparison between groups on secondary outcome measures.

Outcome	Group	T0 Mean	T1 Mean	T2 Mean	Time	Group × Time	Pairwise Post Hoc Comparisons over Time Mean Difference [95% CI]
		(SD)	(SD)	(SD)	*p* (η^2^p)	*p* (η^2^p)	
PBS	MIG	50.06 (5.58)	52.88 (3.46)	52.81 (3.41)	0.006 (0.110)	0.045 (0.097)	T1–T0: 2.81 (0.68–4.94); T2–T0: 2.75 (0.68–4.82)
	Psychology	52.56 (3.45)	52.22 (2.78)	52.89 (3.91)			NS
	Physiotherapy	50.44 (5.37)	50.94 (4.48)	51.75 (4.63)			NS
VABS-II	MIG	76.75 (11.48)	78.6 (11.37)	78 (5.78)	0.710 (0.007)	0.963 (0.006)	NS
	Psychology	73.65 (9.37)	73.88 (7.11)	74.29 (10.15)			NS
	Physiotherapy	75.27 (7.49)	75.2 (6.84)	76.4 (5.80)			NS
DCDq	MIG	38.75 (7.69)	41.35 (14.14)	43.55 (11.18)	0.533 (0.011)	0.340 (0.039)	NS
	Psychology	29.5 (18.44)	32.18 (19.77)	33.41 (20.44)			NS
	Physiotherapy	38.56 (10.26)	39.5 (15.29)	34.5 (18.29)			NS
PROC—expressive language	MIG	57.21 (8.23)	67.16 (4.66)	68.58 (5.10)	<0.001 (0.368)	0.033 (0.114)	T1–T0: 9.95 (5.04–14.86); T2–T0: 11.37 (6.01–16.72)
	Psychology	58.11 (7.41)	61.26 (6.80)	61.26 (7.56)			NS
	Physiotherapy	52 (15.54)	57.8 (7.47)	60.67 (8.72)			T1–T0: 5.80 (0.27–11.33); T2–T0: 8.67 (2.64–14.69)
PROC—verbal comprehension	MIG	52.95 (10.81)	60.05 (5.99)	59.53 (1.5)	0.004 (0.126)	0.306 (0.047)	T1–T0: 7.11 (1.33–12.88); T2–T0: 6.58 (0.13–13.02)
	Psychology	57.47 (6.92)	59.37 (2.31)	58 (5.2)			NS
	Physiotherapy	52.6 (16.17)	57.13 (5.98)	57.67 (4.03)			NS
PROC—Cognitive development	MIG	50.89 (14.06)	60.95 (8.51)	65.16 (6.48)	<0.001 (0.332)	0.072 (0.082)	T1–T0: 10.05 (3.24–16.87); T2–T0: 14.26 (6.24–22.29)
	Psychology	52.37 (10.71)	63.63 (7.59)	64.32 (7.36)			T1–T0: 11.26 (4.45–18.08); T2–T0: 11.95 (3.92–19.98)
	Physiotherapy	49.6 (18.63)	49.73 (13.19)	59.6 (13.53)			T2–T0: 10.00 (0.96–19.04)
ABFW—communicative acts	MIG	36.37 (8.41)	49.63 (4.62)	46.74 (8.83)	<0.001 (0.278)	0.007 (0.128)	T1–T0: 13.26 (7.67–18.85); T2–T0: 10.37 (5.24–15.50)
	Psychology	34.53 (13.35)	37.84 (13.80)	37.79 (11.81)			NS
	Physiotherapy	31.63 (11.28)	35.81 (12.70)	36.56 (14.46)			NS

Legend: PBS: Pediatric Balance Scale; VABS-II—Vineland Adaptive Behavior Scales—Second Edition; DCDq—Developmental Coordination Disorder Questionnaire; PROC: Behavioral Observation Protocol; ABFW: Child Language Test; MIG: Global Integration Method; T0 = baseline; T1 = post-intervention; T2 = 3-month follow-up; NS = non-significant; CI = 95% confidence interval. Vineland and DCDQ did not show evidence of differential effects between interventions. For cognitive development (PROC), a significant main effect of time was also identified (F(1.564, 78.221) = 24.828; *p* = 0.001; η^2^p = 0.332), with no significant time × group interaction. These findings indicate overall improvement across time points in all groups, with no statistically significant differences in trajectories between interventions.

## Data Availability

The data presented in this study are available on request from the corresponding author. The data are not publicly available due to privacy restrictions.
